# Supplementary material for: Shared fate was associated with sustained cooperation during the COVID-19 pandemic
Source: PLoS One. 2024 Sep 26;19(9):e0307829. doi: 10.1371/journal.pone.0307829 (PMC11426462; doi:10.1371/journal.pone.0307829)
Supplement: S1 File — (DOCX) [file pone.0307829.s001.docx]

**Supplemental Information**

**Shared Fate was Associated with Sustained Cooperation During the COVID-19 Pandemic**

[author information redacted for review]

*PLOS ONE*

**S1. Supplemental Method.**

**Table S1. Number of participants from each country represented in the sample.**

**S2. Supplemental Analyses.**

**S2.1. Perception of COVID-19 risk**

**Table S2. GLMMs predicting perceived risk of infection.**

**S2.2. Cooperation with neighbors and with all of humanity**

**Table S3. GLMMs predicting willingness to help a neighbor.**

**Table S4. GLMMs predicting need-based helping attitude toward neighbors.**

**Table S5. GLMMs predicting willingness to help a person from a different country.**

**Table S6. GLMMs predicting need-based helping attitude toward a person from a different country.**

**S2.3. Interdependence with neighbors and all of humanity**

**Table S7. GLMMs predicting emotional shared fate with neighbors.**

**Table S8. GLMMs predicting perceived shared fate with neighbors.**

**Table S9. GLMMs predicting emotional shared fate with all of humanity.**

**Table S10. GLMMs predicting perceived shared fate with all of humanity**

**Table S11. Summary of the changes in perceived interdependence from March to August 2020.**

**S2.4. Cooperation and interdependence with neighbors vs. humanity**

**Table S12. Cooperation between neighbors and a person from a different country.**

**Table S13. Perceived interdependence between neighbors and all of humanity.**

**S1. Supplemental Method.**

**Table S1. Number of participants from each country represented in the sample.**

| Country | *n* | % |
| --- | --- | --- |
| England | 188 | 18.7 |
| Portugal | 153 | 15.2 |
| Poland | 126 | 12.5 |
| US | 87 | 8.6 |
| Canada | 50 | 5 |
| Spain | 47 | 4.7 |
| Greece | 45 | 4.5 |
| UK | 44 | 4.4 |
| Mexico | 35 | 3.5 |
| Scotland | 30 | 3 |
| Italy | 28 | 2.8 |
| Hungary | 22 | 2.2 |
| Australia | 16 | 1.6 |
| Wales | 14 | 1.4 |
| Czech Republic | 11 | 1.1 |
| France | 10 | 1 |
| Germany | 9 | 0.9 |
| Netherlands | 9 | 0.9 |
| Slovenia | 8 | 0.8 |
| Israel | 7 | 0.7 |
| Estonia | 6 | 0.6 |
| Chile | 6 | 0.6 |
| Ireland | 6 | 0.6 |
| Belgium | 6 | 0.6 |
| Sweden | 5 | 0.5 |
| Latvia | 5 | 0.5 |
| Denmark | 5 | 0.5 |
| Finland | 5 | 0.5 |
| Austria | 4 | 0.4 |
| Switzerland | 4 | 0.4 |
| Norway | 4 | 0.4 |
| New Zealand | 3 | 0.3 |
| Luxembourg | 2 | 0.2 |
| Angola | 1 | 0.1 |
| South Korea | 1 | 0.1 |
| Ecuador | 1 | 0.1 |
| Turkey | 1 | 0.1 |
| Northern Ireland | 1 | 0.1 |
| NA | 1 | 0.1 |

**S2. Supplemental Analyses.**

**S2.1. Perception of COVID-19 risk.**

After testing the effect of time on perceived risk of COVID-19 infection, we separately test a quadratic effect of time model, a COVID-19 prevalence model, and a combined model. In the quadratic effect of time model, we include the effect of time and a time *×* time interaction. In the prevalence of COVID-19 model, we include prevalence of COVID-19 and its interaction with time at both Level-1 and Level-2. In the combined model we include a time *×* time *×* COVID-19 prevalence interaction at both Level-1 and Level-2.

Perceived risk of COVID-19 infection decreased over time (*b* = -0.01, SE = 0.004, CI_95%_ [-0.02, -0.0006]), leading to a decrease of *b* = -0.11 (SE = 0.05, CI_95%_ [-0.22, -0.01]) by time 14. Table S2 shows results for the quadratic effect of time, COVID-19 prevalence model, and the COVID-19 prevalence and quadratic effect of time model. The COVID-19 prevalence and quadratic effect of time model improved fit relative to an intercepts-only model (Δ-2LL 𝝌(10)^2^ = 583.78, *p* < 0.001), accounting for 67.60% of the between-person (i.e., Level-2) variance, and 6.22% of the within-person (i.e., Level-1 variance) (ICC = 0.35).

**Table S2. GLMMs predicting perceived risk of infection.**

|  | Time^2^ | | | | COVID prevalence | | | | Time^2^ and COVID | | | |
| --- | --- | --- | --- | --- | --- | --- | --- | --- | --- | --- | --- | --- |
| Fixed effects | *b* | *p* | 95% CI | | *b* | *p* | 95% CI | | *b* | *p* | 95% CI | |
| Time | **-.122** | **<.001** | **-.153** | **-.091** | -.010 | .405 | -.034 | 0.014 | **-.174** | **.0003** | **-.268** | **-.080** |
| Time *×* time | **.006** | **<.001** | **.004** | **.008** |  |  |  |  | **.012** | **<.001** | **.006** | **.018** |
| *Level-2* |  |  |  |  |  |  |  |  |  |  |  |  |
| COVID prevalence |  |  |  |  | .040 | .310 | -.037 | 0.117 | .029 | .662 | -.102 | .160 |
| Time *×* COVID |  |  |  |  | .004 | .449 | -.006 | 0.013 | **.053** | **.003** | **.019** | **.088** |
| Time *×* time *×* COVID |  |  |  |  |  |  |  |  | **-.003** | **.004** | **-.005** | **-.001** |
| *Level-1* |  |  |  |  |  |  |  |  |  |  |  |  |
| COVID prevalence |  |  |  |  | .040 | .197 | -.021 | 0.101 | **.376** | **<.001** | **.271** | **.480** |
| Time *×* COVID |  |  |  |  | -.019 | .090 | -.040 | 0.003 | **-.159** | **<.001** | **-.210** | **-.107** |
| Time *×* time *×* COVID |  |  |  |  |  |  |  |  | **.006** | **.013** | **.001** | **.011** |
| Random effects | 𝝉 | *SE* | *p* |  | 𝝉 | *SE* | *p* |  | 𝝉 | *SE* | *p* |  |
| Intercept | .538 | .034 | <.001 |  | .580 | .035 | <.001 |  | .579 | .036 | <.001 |  |
| Time | .013 | .001 | <.001 |  | .008 | .001 | <.001 |  | .009 | .001 | <.001 |  |
| COVID prevalence |  |  |  |  | .072 | .016 | <.001 |  | .126 | .024 | <.001 |  |
| σ^2^ | 23212.84 | | |  | 22202.68 | | |  | 21822.87 | | |  |

*Note*. Time^2^ model (Obs. = 9063, *N* = 995), COVID prevalence model (Obs. = 8814, *N* = 932), Time^2^ and COVID prevalence model (Obs. = 8814, *N* = 932), ICC = 0.35. Numbers in bold show statistically significant fixed effects. Perceived risk of infection was measured as *How likely do you think it is that you will become infected with COVID19?* (1 = *not at all*, 7 = *extremely*).

**S2.2. Cooperation with neighbors and with all of humanity.**

After testing the effect of time and the time *×* time interaction on inclinations to cooperate, we separately test a disease avoidance model and a shared fate model. In the disease avoidance model, we include prevalence of COVID-19, perceived risk of infection, and each covariate’s interaction with time at both Level-1 and Level-2. In the shared fate model, we include emotional shared fate, perceived shared fate, and each covariate’s interaction with time at both Level-1 and Level-2. In the disease avoidance and shared fate model, we retain statistically significant covariates from both models.

**S2.2.1. Willingness to help a neighbor by allowing them to move in for a week.**

Willingness to help neighbors decreased over time (*b* = -0.05, SE = 0.004, CI_95%_ [-0.06, -0.04]), leading to a decrease in willingness to help of *b* = -0.65 (SE = 0.06, CI_95%_ [-0.77, -0.54]) by time 14. We did not find a time *×* time interaction (*b* = 0.002, SE = 0.001, CI_95%_ [-0.0002, 0.004]), indicating that willingness to help neighbors decreased linearly over time. Table S3 shows results for the disease avoidance, shared fate, and the combined model. Although we find a statistically significant time *×* perceived risk (Level-1) interaction in the combined model (*b* = 0.01, SE = 0.004, CI_95%_ [0.003, 0.02]), including this interaction leads to worse model fit relative to an intercepts-only model (Δ-2LL = 8.66). When removing the time *×* perceived risk (Level-1) interaction, the combined model improves model fit relative to an intercepts-only model (Δ-2LL 𝝌(14)^2^ = 33.84, *p* < 0.01), accounting for 78.55% of the between-person (i.e., Level-2) variance, but none of the within-person (i.e., Level-1) variance (ICC = 0.49).

**Table S3. GLMMs predicting willingness to help a neighbor.**

|  | Disease avoidance | | | | Shared fate | | | | Disease avoidance  and shared fate | | | |
| --- | --- | --- | --- | --- | --- | --- | --- | --- | --- | --- | --- | --- |
| Fixed effects | *b* | *p* | 95% CI | | *b* | *p* | 95% CI | | *b* | *p* | 95% CI | |
| Time | **-.031** | **.026** | **-.058** | **-.004** | **-.124** | **<.001** | **-.150** | **-.099** | **-.056** | **.001** | **-.088** | **-.023** |
| *Level-2* |  |  |  |  |  |  |  |  |  |  |  |  |
| COVID prevalence | .040 | .350 | -.044 | .125 |  |  |  |  | .038 | .379 | -.046 | .121 |
| Perceived risk | -.007 | .877 | -.093 | .079 |  |  |  |  | -.034 | .436 | -.118 | .051 |
| Perceived SF |  |  |  |  | .071 | .207 | -.039 | .181 | .095 | .098 | -.017 | .206 |
| Emotional SF |  |  |  |  | **.307** | **<.001** | **.193** | **.420** | **.280** | **<.001** | **.169** | **.390** |
| Time *×* COVID | **.026** | **<.001** | **.013** | **.039** |  |  |  |  | **-.022** | **.001** | **-.034** | **-.009** |
| Time *×* Risk | **.016** | **.012** | **.003** | **.028** |  |  |  |  | **-.022** | **.001** | **-.034** | **-.009** |
| Time *×* PSF |  |  |  |  | **.027** | **.002** | **.010** | **.044** | **.032** | **<.001** | **.017** | **.048** |
| Time *×* ESF |  |  |  |  | .014 | .106 | -.003 | .032 |  |  |  |  |
| *Level-1* |  |  |  |  |  |  |  |  |  |  |  |  |
| COVID prevalence | .044 | .206 | -.024 | .113 |  |  |  |  | .014 | .688 | -.056 | .084 |
| Perceived risk | -.029 | .315 | -.086 | .028 |  |  |  |  | .003 | .812 | -.023 | .029 |
| Perceived SF |  |  |  |  | .038 | .200 | -.020 | .095 | **.071** | **<.001** | **.042** | **.099** |
| Emotional SF |  |  |  |  | **.093** | **.002** | **.035** | **.151** | **.044** | **.003** | **.015** | **.073** |
| Time *×* COVID | **-.036** | **.004** | **-.061** | **-.012** |  |  |  |  | **-.047** | **.0003** | **-.073** | **-.022** |
| Time *×* Risk | **.010** | **.009** | **.002** | **.017** |  |  |  |  |  |  |  |  |
| Time *×* PSF |  |  |  |  | .002 | .584 | -.005 | .010 |  |  |  |  |
| Time *×* ESF |  |  |  |  | -.004 | .295 | -.011 | .003 |  |  |  |  |
| Random effects | 𝝉 | *SE* | *p* |  | 𝝉 | *SE* | *p* |  | 𝝉 | *SE* | *p* |  |
| Intercept | .742 | .056 | <.001 |  | .683 | .069 | <.001 |  | .673 | .083 | <.001 |  |
| Time | .015 | .002 | <.001 |  | .016 | .002 | <.001 |  | .014 | .003 | <.001 |  |
| σ^2^ | 18936.54 | | |  | 22280.10 | | |  | 20386.14 | | |  |

*Note.* Disease avoidance model (Obs. = 8247, *N* = 926), Shared fate model (Obs. = 9031, *N* = 998), Disease avoidance and shared fate model (Obs. = 8223, *N* = 926), ICC = 0.49. Numbers in bold show statistically significant fixed effects. Willingness to help neighbors was measured as *Someone from your neighborhood is having their house fixed, so it isn't livable. How willing would you be to let them move into your house for a week?* (1 = not at all, 7 = very willing).

**S2.2.2. Need-based helping attitude toward neighbors.**

Need-based attitude towards neighbors decreased over time (*b* = -0.04, SE = 0.005, CI_95%_ [-0.05, -0.03]), leading to a decrease of *b* = -0.52 (SE = 0.06, CI_95%_ [-0.65, -0.39]) by time 14. We did not find a time *×* time interaction (*b* = -0.002, SE = 0.001, CI_95%_ [-0.003, 0.0001]), indicating that need-based attitude towards neighbors decreased linearly over time. Table S4 shows results for the disease avoidance, shared fate, and the combined model. The disease avoidance and shared fate model improved fit relative to an intercepts-only model (Δ-2LL 𝝌(14)^2^ = 2035.18, *p* < 0.001), accounting for 49.58% of the between-person (i.e., Level-2) variance, and 13.63% of the within-person (i.e., Level-1) variance (ICC = 0.38).

**Table S4. GLMMs predicting need-based helping attitude toward neighbors.**

|  | Disease avoidance | | | | Shared fate | | | | Disease avoidance  and shared fate | | | |
| --- | --- | --- | --- | --- | --- | --- | --- | --- | --- | --- | --- | --- |
| Fixed effects | *b* | *p* | 95% CI | | *b* | *p* | 95% CI | | *b* | *p* | 95% CI | |
| Time | .023 | .144 | -.008 | .055 | .002 | .752 | -.009 | .012 | -.003 | .601 | -.016 | .009 |
| *Level-2* |  |  |  |  |  |  |  |  |  |  |  |  |
| COVID prevalence | **.108** | **.014** | **.022** | **.194** |  |  |  |  | **.171** | **<.001** | **.093** | **.249** |
| Perceived risk | .075 | .090 | -.012 | .162 |  |  |  |  | **.111** | **.021** | **.017** | **.206** |
| Perceived SF |  |  |  |  | **-.139** | **.011** | **-.245** | **-.032** | **-.174** | **.003** | **-.289** | **-.059** |
| Emotional SF |  |  |  |  | **.571** | **<.001** | **.464** | **.678** | **.668** | **<.001** | **.532** | **.804** |
| Time *×* COVID | .007 | .267 | -.005 | .018 |  |  |  |  |  |  |  |  |
| Time *×* Risk | **-.012** | **.045** | **-.024** | **-.0003** |  |  |  |  | **-.016** | **.002** | **-.026** | **-.006** |
| Time *×* PSF |  |  |  |  | .008 | .310 | -.007 | .023 |  |  |  |  |
| Time *×* ESF |  |  |  |  | **.024** | **.002** | **.009** | **.039** | **.033** | **<.001** | **.022** | **.044** |
| *Level-1* |  |  |  |  |  |  |  |  |  |  |  |  |
| COVID prevalence | **-.181** | **<.001** | **-.267** | **-.094** |  |  |  |  | **-.281** | **<.001** | **-.349** | **-.212** |
| Perceived risk | -.005 | .872 | -.066 | .056 |  |  |  |  |  |  |  |  |
| Perceived SF |  |  |  |  | -.050 | .121 | -.114 | .013 | -.039 | .248 | -.104 | .027 |
| Emotional SF |  |  |  |  | **.130** | **<.001** | **.071** | **.189** | **.139** | **<.001** | **.111** | **.167** |
| Time *×* COVID | .003 | .862 | -.027 | .032 |  |  |  |  |  |  |  |  |
| Time *×* Risk | .001 | .713 | -.006 | .009 |  |  |  |  |  |  |  |  |
| Time *×* PSF |  |  |  |  | **.018** | **<.001** | **.010** | **.026** | **.008** | **.049** | **.00002** | **.016** |
| Time *×* ESF |  |  |  |  | .002 | .688 | -.006 | .009 |  |  |  |  |
| Random effects | 𝝉 | *SE* | *p* |  | 𝝉 | *SE* | *p* |  | 𝝉 | *SE* | *p* |  |
| Intercept | .632 | .046 | <.001 |  | .486 | .036 | <.001 |  | 1.012 | .077 | <.001 |  |
| Time | .012 | .001 | <.001 |  | .011 | .0001 | <.001 |  | .006 | .001 | <.001 |  |
| COVID prevalence | .221 | .042 | <.001 |  |  |  |  |  | .119 | .028 | <.001 |  |
| Perceived SF |  |  |  |  | .015 | .004 | .001 |  | .042 | .008 | <.001 |  |
| σ^2^ | 18111.71 | | |  | 20398.24 | | |  | 18561.14 | | |  |

*Note.* Disease avoidance model (Obs. = 8253, *N* = 926), Shared fate model (Obs. = 9036, *N* = 998), Disease avoidance and shared fate model (Obs. = 8782, *N* = 927), ICC = 0.38. Numbers in bold show statistically significant fixed effects. Need-based helping attitude toward neighbors was measured as *helping someone from my neighborhood when they are in need is the right thing to do* (1 = *strongly disagree*, 7 = *strongly agree*).

**S2.2.3. Willingness to help a “displaced person who is not a citizen of your own country” by allowing them to move in for a week.**

We found that willingness to help a person who is not a citizen of your own country decreased over time (*b* = -0.02, SE = 0.005, CI_95%_ [-0.03, -0.01]), leading to a decrease of *b* = -0.25 (SE = 0.07, CI_95%_ [-0.39, -0.11]) by time 14. We did not find a time *×* time interaction (*b* = -0.002, SE = 0.001, CI_95%_ [-0.004, 0.00003]), indicating that willingness to help a person who is not a citizen of your own country decreased linearly over time. Table S5 shows results for the disease avoidance, shared fate, and the combined model. Although we find a statistically significant time *×* perceived risk (Level-1) interaction in the combined model (*b* = 0.03, SE = 0.004, CI_95%_ [0.02, 0.03]), including this interaction leads to worse model fit relative to an intercepts-only model (Δ-2LL = 605.37). When removing the time *×* perceived risk (Level-1) interaction, the combined model improves model fit relative to an intercepts-only model (Δ-2LL 𝝌(12)^2^ = 147.18, *p* < 0.001), accounting for 78.96% of the between-person (i.e., Level-2) variance, and 0.20% of the within-person (i.e., Level-1) variance (ICC = 0.51).

**Table S5. GLMMs predicting willingness to help a person from a different country.**

|  | Disease avoidance | | | | Shared fate | | | | Disease avoidance  and shared fate | | | |
| --- | --- | --- | --- | --- | --- | --- | --- | --- | --- | --- | --- | --- |
| Fixed effects | *b* | *p* | 95% CI | | *b* | *p* | 95% CI | | *b* | *p* | 95% CI | |
| Time | **-.083** | **.0003** | **-.128** | **-.039** | **-.097** | **<.001** | **-.115** | **-.078** | **-.073** | **.011** | **-.129** | **-.016** |
| *Level-2* |  |  |  |  |  |  |  |  |  |  |  |  |
| COVID prevalence | .054 | .236 | -.035 | .142 |  |  |  |  | .066 | .152 | -.024 | .155 |
| Perceived risk | .030 | .531 | -.063 | .122 |  |  |  |  | -.001 | .987 | -.081 | .080 |
| Perceived SF |  |  |  |  | .049 | .398 | -.064 | .162 | .068 | .259 | -.050 | .185 |
| Emotional SF |  |  |  |  | **.207** | **.0004** | **.093** | **.322** | **.149** | **.009** | **.036** | **.260** |
| Time *×* COVID | **-.016** | **.020** | **-.029** | **-.003** |  |  |  |  | **-.017** | **.046** | **-.034** | **-.0003** |
| Time *×* Risk | -.008 | .273 | -.021 | .006 |  |  |  |  |  |  |  |  |
| Time *×* PSF |  |  |  |  | **.022** | **.018** | **.004** | **.041** | **.029** | **.011** | **.007** | **.052** |
| Time *×* ESF |  |  |  |  | .001 | .908 | -.017 | .019 |  |  |  |  |
| *Level-1* |  |  |  |  |  |  |  |  |  |  |  |  |
| COVID prevalence | **.114** | **.003** | **.040** | **.188** |  |  |  |  | **.116** | **.004** | **.036** | **.196** |
| Perceived risk | **-.058** | **.051** | **-.116** | **.0002** |  |  |  |  | .009 | .521 | -.018 | .036 |
| Perceived SF |  |  |  |  | **.077** | **.008** | **.020** | **.133** | **.048** | **.001** | **.020** | **.075** |
| Emotional SF |  |  |  |  | .015 | .619 | -.044 | .074 |  |  |  |  |
| Time *×* COVID | **-.051** | **.0001** | **-.076** | **-.025** |  |  |  |  | **-.041** | **.007** | **-.071** | **-.011** |
| Time *×* Risk | **.022** | **<.001** | **.014** | **.029** |  |  |  |  |  |  |  |  |
| Time *×* PSF |  |  |  |  | -.003 | .403 | -.011 | .004 |  |  |  |  |
| Time *×* ESF |  |  |  |  | -.002 | .552 | -.010 | .005 |  |  |  |  |
| Random effects | 𝝉 | *SE* | *p* |  | 𝝉 | *SE* | *p* |  | 𝝉 | *SE* | *p* |  |
| Intercept | .738 | .096 | <.001 |  | .748 | .057 | <.001 |  | .727 | .168 | <.001 |  |
| Time | .020 | .004 | <.001 |  | .020 | .002 | <.001 |  | .022 | .012 | .034 |  |
| σ^2^ | 18988.12 | | |  | 19769.6 | | |  | 18588.65 | | |  |

*Note.* Disease avoidance model (Obs. = 8246, *N* = 926), Shared fate model (Obs. = 9039, *N* = 998), Disease avoidance and shared fate model (Obs. = 8232, *N* = 926), ICC = 0.51. Numbers in bold show statistically significant fixed effects. Willingness to help a person from a different country was measured as *A displaced person who is not a citizen of your own country is having their house fixed, so it isn't livable. How willing would you be to let them move into your house for a week?* (1 = *not at all*, 7 = *very willing*).

**S2.2.4. Need-based helping attitude towards a person from a different country.**

Need-based helping attitude towards a person who is not a citizen of your own country decreased over time (*b* = -0.02, SE = 0.004, CI_95%_ [-0.03, -0.02]), leading to a decrease of *b* = -0.34 (SE = 0.06, CI_95%_ [-0.46, -0.22]) by time 14. Although we found a time *×* time interaction (*b* = 0.004, SE = 0.001, CI_95%_ [0.002, 0.006]), this interaction led to a worse fitting model compared to an intercepts-only model (Δ-2LL = 98.89), indicating that the linear effect of time was a better fit to the data than the quadratic effect of time.

Table S6 shows results for the disease avoidance, shared fate, and the combined model. The disease avoidance and shared fate model improved fit relative to an intercepts-only model (Δ-2LL 𝝌(14)^2^ = 2565.10, *p* < 0.001), accounting for 72.06% of the between-person (i.e., Level-2) variance, and 14.41% of the within-person (i.e., Level-1) variance (ICC = 0.38).

**Table S6. GLMMs predicting need-based helping attitude toward a person from a different country.**

|  | Disease avoidance | | | | Shared fate | | | | Disease avoidance  and shared fate | | | |
| --- | --- | --- | --- | --- | --- | --- | --- | --- | --- | --- | --- | --- |
| Fixed effects | *b* | *p* | 95% CI | | *b* | *p* | 95% CI | | *b* | *p* | 95% CI | |
| Time | **.052** | **.001** | **.022** | **.081** | .006 | .252 | -.004 | .017 | **.045** | **.002** | **.017** | **.073** |
| *Level-2* |  |  |  |  |  |  |  |  |  |  |  |  |
| COVID prevalence | **.126** | **.006** | **.037** | **.215** |  |  |  |  | **.111** | **.002** | **.040** | **.183** |
| Perceived risk | **.105** | **.019** | **.018** | **.193** |  |  |  |  | **.074** | **.045** | **.002** | **.147** |
| Perceived SF |  |  |  |  | -.051 | .340 | -.156 | .054 | -.070 | .250 | -.188 | .049 |
| Emotional SF |  |  |  |  | **.507** | **<.001** | **.403** | **.611** | **.511** | **<.001** | **.396** | **.626** |
| Time *×* COVID | -.009 | .160 | -.021 | .003 |  |  |  |  |  |  |  |  |
| Time *×* Risk | .002 | .774 | -.011 | .014 |  |  |  |  |  |  |  |  |
| Time *×* PSF |  |  |  |  | **-.019** | **.014** | **-.035** | **-.004** | **-.021** | **.027** | **-.040** | **-.002** |
| Time *×* ESF |  |  |  |  | **.040** | **<.001** | **.025** | **.056** | **.036** | **.0001** | **.018** | **.055** |
| *Level-1* |  |  |  |  |  |  |  |  |  |  |  |  |
| COVID prevalence | **-.146** | **.0004** | **-.226** | **-.066** |  |  |  |  | **-.149** | **.0001** | **-.225** | **-.072** |
| Perceived risk | .002 | .956 | -.061 | .065 |  |  |  |  |  |  |  |  |
| Perceived SF |  |  |  |  | -.019 | .521 | -.076 | .038 |  |  |  |  |
| Emotional SF |  |  |  |  | **.101** | **.001** | **.042** | **.161** | **.108** | **<.001** | **.076** | **.140** |
| Time *×* COVID | **-.034** | **.013** | **-.062** | **-.007** |  |  |  |  | **-.037** | **.004** | **-.062** | **-.011** |
| Time *×* Risk | -.002 | .674 | -.009 | .006 |  |  |  |  |  |  |  |  |
| Time *×* PSF |  |  |  |  | .005 | .172 | -.002 | .012 |  |  |  |  |
| Time *×* ESF |  |  |  |  | -.001 | .742 | -.009 | .006 |  |  |  |  |
| Random effects | 𝝉 | *SE* | *p* |  | 𝝉 | *SE* | *p* |  | 𝝉 | *SE* | *p* |  |
| Intercept | .652 | .049 | <.001 |  | .535 | .039 | <.001 |  | .548 | .054 | <.001 |  |
| Time | .016 | .002 | <.001 |  | .012 | .001 | <.001 |  | .018 | .002 | <.001 |  |
| Risk | .033 | .011 | .001 |  |  |  |  |  | .0005 | .005 | .458 |  |
| COVID prevalence | .118 | .036 | .0006 |  |  |  |  |  | .062 | .029 | .017 |  |
| Perceived SF |  |  |  |  | .018 | .007 | .005 |  |  |  |  |  |
| Emotional SF |  |  |  |  | .020 | .008 | .004 |  | .046 | .014 | .0004 |  |
| σ^2^ | 18609.27 | | |  | 20862.36 | | |  | 18707.1 | | |  |

*Note.* Disease avoidance model (Obs. = 8251, *N* = 926), Shared fate model (Obs. = 9043, *N* = 998), Disease avoidance and shared fate model (Obs. = 8245, *N* = 926), ICC = 0.38. Numbers in bold show statistically significant fixed effects. Need-based helping attitude towards a person from a different country was measured as *Helping a displaced person who is not a citizen of your own country when they are in need is the right thing to do* (1 = *strongly disagree*, 7 = *strongly agree*).

**S2.3. Perceived interdependence with neighbors and all of humanity.**

After testing the effect of time and the time *×* time interaction on perceived interdependence, we separately test a prevalence of COVID-19 model and a perceived risk of infection model. In the prevalence of COVID-19 model, we include prevalence of COVID-19 and its interaction with time at both Level-1 and Level-2. In the perceived risk of infection model, we include perceived risk of infection and its interaction with time at both Level-1 and Level-2. In the disease avoidance model, we retain statistically significant covariates from both models.

**S2.3.1. Emotional shared fate - “When my neighborhood succeeds, I feel good”.**

Emotional shared fate with neighbors increased over time (*b* = 0.01, SE = 0.004, CI_95%_ [0.003, 0.02]), leading to an increase of *b* = 0.14 (SE = 0.05, CI_95%_ [0.04, 0.25]) by time 14. The time *×* time interaction was statistically significant (*b* = -0.002, SE = 0.001, CI_95%_ [-0.004, -0.001]). However, including the time *×* time interaction led to worse model fit relative to an intercepts-only model (Δ-2LL = 853.60), indicating that the linear effect of time was a better fit to the data than the quadratic effect of time. Table S7 shows the results for the COVID prevalence, perceived risk, and disease avoidance model. The disease avoidance model improved fit relative to an intercepts-only model (Δ-2LL 𝝌(9)^2^ = 2715.37, *p* < 0.001), accounting for 22.20% of the between-person (i.e., Level-2) variance, and 16.11% of the within-person (i.e., Level-1) variance (ICC = 0.44).

**Table S7. GLMMs predicting emotional shared fate with neighbors.**

|  | COVID prevalence | | | | Perceived risk | | | | Disease avoidance | | | |
| --- | --- | --- | --- | --- | --- | --- | --- | --- | --- | --- | --- | --- |
| Fixed effects | *b* | *p* | 95% CI | | *b* | *p* | 95% CI | | *b* | *p* | 95% CI | |
| Time | **.002** | **.828** | **-.020** | **.024** | **.085** | **<.001** | **.044** | **.126** | .021 | .073 | -.002 | .044 |
| *Level-2* |  |  |  |  |  |  |  |  |  |  |  |  |
| COVID prevalence | **.151** | **.005** | **.046** | **.256** |  |  |  |  | .017 | .744 | -.084 | .118 |
| Perceived risk |  |  |  |  | .047 | .274 | -.037 | .130 | .072 | .221 | -.044 | .188 |
| Time *×* COVID | -.005 | .275 | -.014 | .004 |  |  |  |  |  |  |  |  |
| Time *×* Risk |  |  |  |  | **-.044** | **.0001** | **-.066** | **-.021** | -.007 | .106 | -.017 | .002 |
| *Level-1* |  |  |  |  |  |  |  |  |  |  |  |  |
| COVID prevalence | **.180** | **<.001** | **.115** | **.244** |  |  |  |  | .065 | .143 | -.022 | .151 |
| Perceived risk |  |  |  |  | .014 | .598 | -.039 | .068 | **.029** | **.030** | **.003** | **.055** |
| Time *×* COVID | **-.020** | **.056** | **-.040** | **.001** |  |  |  |  | **-.033** | **.004** | **-.056** | **-.011** |
| Time *×* Risk |  |  |  |  | .003 | .421 | -.004 | .010 |  |  |  |  |
| Random effects | 𝝉 | *SE* | *p* |  | 𝝉 | *SE* | *p* |  | 𝝉 | *SE* | *p* |  |
| Intercept | 1.699 | .082 | <.001 |  | .715 | .064 | <.001 |  | 2.024 | .103 | <.001 |  |
| Time | .004 | .0006 | <.001 |  | .014 | .004 | .001 |  | .003 | .0006 | <.001 |  |
| COVID prevalence | .108 | .022 | .001 |  |  |  |  |  | .240 | .042 | <.001 |  |
| σ^2^ | 19696.19 | | |  | 21136.69 | | |  | 18103.1 | | |  |

*Note.* COVID-19 prevalence model (Obs. = 8807, *N* = 932), Perceived risk of infection model (Obs. = 8475, *N* = 988), Disease avoidance model (Obs. = 8247, *N* = 926), ICC = 0.44. Numbers in bold show statistically significant fixed effects. Emotional shared fate with neighbors was measured as *When my neighborhood succeeds I feel good* (1 = *do not agree at all*, 7 = *strongly agree*).

**S2.3.2. Perceived shared fate - “My neighborhood and I rise and fall together”.**

Perceived shared fate with neighbors increased over time (*b* = 0.05, SE = 0.005, CI_95%_ [0.04, 0.06]), leading to an increase of *b* = 0.66 (SE = 0.06, CI_95%_ [0.53, 0.79]) by time 14. We did not find a time *×* time interaction (*b* = 0.001, SE = 0.001, CI_95%_ [-0.0003, 0.003]), indicating that perceived shared fate with neighbors increased linearly over time.

Table S8 shows the results for the COVID-19 prevalence model, and the perceived risk of infection model. Perceived risk of infection was not associated with perceived shared fate with neighbors at time 1 or over time, we thus focus on the COVID-19 prevalence model. The COVID prevalence model improved fit relative to an intercepts-only model (Δ-2LL 𝝌(7)^2^ = 1347.35, *p* < 0.001), accounting for 27.58% of the between-person (i.e., Level-2) variance, and 22.15% of the within-person (i.e., Level-1) variance (ICC = 0.40).

**Table S8. GLMMs predicting perceived shared fate with neighbors.**

|  | COVID prevalence | | | | Perceived risk | | | |
| --- | --- | --- | --- | --- | --- | --- | --- | --- |
| Fixed effects | *b* | *p* | 95% CI | | *b* | *p* | 95% CI | |
| Time | **.035** | **.003** | **.012** | **.058** | **.020** | **.0001** | **.010** | **.030** |
| *Level-2* |  |  |  |  |  |  |  |  |
| COVID prevalence | **-.167** | **.002** | **-.272** | **-.063** |  |  |  |  |
| Time *×* COVID | **.026** | **<.001** | **.016** | **.036** |  |  |  |  |
| Perceived risk |  |  |  |  | .065 | .112 | -.015 | .144 |
| Time *×* Risk |  |  |  |  | .007 | .228 | -.004 | .017 |
| *Level-1* |  |  |  |  |  |  |  |  |
| COVID prevalence | **.376** | **<.001** | **.304** | **.448** |  |  |  |  |
| Time *×* COVID | **-.038** | **.001** | **-.059** | **-.017** |  |  |  |  |
| Perceived risk |  |  |  |  | .030 | .272 | -.024 | .084 |
| Time *×* Risk |  |  |  |  | -.003 | .344 | -.010 | .004 |
| Random effects | 𝝉 | *SE* | *p* |  | 𝝉 | *SE* | *p* |  |
| Intercept | 1.597 | .081 | <.001 |  | .726 | .038 | <.001 |  |
| Time | .005 | .001 | <.001 |  | .012 | .001 | <.001 |  |
| COVID prevalence | .176 | .031 | .001 |  |  |  |  |  |
| σ^2^ | 21358.72 | | |  | 21419.15 | | |  |

*Note.* COVID-19 prevalence model (Obs. = 8796, *N* = 932), Perceived risk of infection model (Obs. = 8464, *N* = 988), ICC = 0.40. Numbers in bold show statistically significant fixed effects. Perceived shared fate with neighbors was measured as *My neighborhood and I rise and fall together* (1 = *do not agree at all*, 7 = *strongly agree*).

**S2.3.3. Emotional shared fate - “When all of humanity succeeds, I feel good”.**

Emotional shared fate with all of humanity decreased over time (*b* = -0.04, SE = 0.004, CI_95%_ [-0.04, -0.03]), leading to a decrease of *b* = -0.48 (SE = 0.05, CI_95%_ [-0.59, -0.37]) by time 14. Although the time *×* time interaction was statistically significant (*b* = -0.005, SE = 0.001, CI_95%_ [-0.007, -0.003]), the interaction led to a worse fitting model relative to an intercepts-only model (Δ-2LL = 139.75), indicating that the linear effect of time was a better fit to the data than the quadratic effect of time.

Table S9 shows results for the COVID-19 prevalence, perceived risk of infection, and disease avoidance model. Though we find a positive effect of perceived risk of infection (Level-1) on emotional shared fate at time 1 in the perceived risk of infection model, this effect did not hold in the disease avoidance model. We therefore ran an additional model (i.e., COVID prevalence 2) in which we removed perceived risk of infection. This model (i.e., COVID prevalence 2) improved fit relative to an intercepts-only model (Δ-2LL 𝝌(6)^2^ = 844.94, *p* < 0.001), accounting for 48.82% of the between-person (i.e., Level-2) variance, and 8.50% of the within-person (i.e., Level-1) variance (ICC = 0.42).

**Table S9. GLMMs predicting emotional shared fate with all of humanity.**

|  | COVID prevalence 1 | | | | Perceived risk | | | | Disease avoidance | | | | COVID prevalence 2 | | | |
| --- | --- | --- | --- | --- | --- | --- | --- | --- | --- | --- | --- | --- | --- | --- | --- | --- |
| Fixed effects | *b* | *p* | 95% CI | | *b* | *p* | 95% CI | | *b* | *p* | 95% CI | | *b* | *p* | 95% CI | |
| Time | -.003 | .824 | -.025 | .020 | -.002 | .653 | -.013 | .008 | .009 | .443 | -.015 | .033 | **.028** | **.017** | **.005** | **.051** |
| *Level-2* |  |  |  |  |  |  |  |  |  |  |  |  |  |  |  |  |
| COVID prevalence | .006 | .921 | -.106 | .117 |  |  |  |  | .025 | .622 | -.073 | .122 | .032 | .449 | -.050 | .113 |
| Time *×* COVID | -.002 | .669 | -.011 | .007 |  |  |  |  |  |  |  |  |  |  |  |  |
| Perceived risk |  |  |  |  | .039 | .388 | -.050 | .129 | .091 | .067 | -.006 | .187 |  |  |  |  |
| Time *×* Risk |  |  |  |  | -.008 | .136 | -.018 | .003 |  |  |  |  |  |  |  |  |
| *Level-1* |  |  |  |  |  |  |  |  |  |  |  |  |  |  |  |  |
| COVID prevalence | -.040 | .263 | -.110 | .030 |  |  |  |  | **-.127** | **.004** | **-.213** | **-.040** | **-.134** | **<.001** | **-.202** | **-.067** |
| Time *×* COVID | **-.036** | **.001** | **-.057** | **-.015** |  |  |  |  | -.019 | .112 | -.043 | .004 | **-.041** | **.0001** | **-.062** | **-.020** |
| Perceived risk |  |  |  |  | **.060** | **.040** | **.003** | **.117** | -.014 | .323 | -.041 | .013 |  |  |  |  |
| Time *×* Risk |  |  |  |  | -.002 | .550 | -.009 | .005 |  |  |  |  |  |  |  |  |
| Random effects | 𝝉 | *SE* | *p* |  | 𝝉 | *SE* | *p* |  | 𝝉 | *SE* | *p* |  | 𝝉 | *SE* | *p* |  |
| Intercept | 1.940 | .102 | <.001 |  | 1.022 | .057 | <.001 |  | 1.736 | .091 | <.001 |  | 1.195 | .061 | <.001 |  |
| Time | .003 | .0007 | <.001 |  | .011 | .001 | .001 |  | .004 | .001 | <.001 |  | .006 | .001 | <.001 |  |
| COVID prevalence | .124 | .032 | .001 |  |  |  |  |  | .174 | .042 | <.001 |  | .085 | .028 | .001 |  |
| σ^2^ | 17272.16 | | |  | 17110.44 | | |  | 16064.5 | | |  | 17494.54 | | |  |

*Note.* COVID-19 prevalence model 1-2 (Obs. = 8810, *N* = 932), Perceived risk of infection model (Obs. = 8475, *N* = 988), Disease avoidance model (Obs. = 8248, *N* = 926), ICC = 0.42. Numbers in bold show statistically significant fixed effects. We used adaptive quadrature as the estimation method for the COVID prevalence 2 model due to convergence issues when applying Laplace approximation as the estimation method. Emotional shared fate with humanity was measured as *When all of humanity succeeds I feel good* (1 = *do not agree at all*, 7 = *strongly agree*).

**S2.3.4. Perceived Shared Fate - “All of humanity and I rise and fall together”.**

Perceived shared fate with all of humanity increased over time (*b* = 0.01, SE = 0.004, CI_95%_ [0.001, 0.02]), leading to an increase of *b* = 0.12 (SE = 0.05, CI_95%_ [0.02, 0.23]) by time 14. We did not find a time *×* time interaction (*b* = -0.001, SE = 0.001, CI_95%_ [-0.003, 0.0005]), indicating that perceived shared fate with humanity increased linearly over time.

Table S10 shows results for the COVID-19 prevalence, and the perceived risk of infection model. COVID-19 prevalence and perceived risk of infection were not associated with perceived shared fate with humanity at time 1 or over time. The linear effect of time alone improved fit relative to an intercepts-only model (Δ-2LL 𝝌(2)^2^ = 299.93, *p* < 0.001), accounting for 26.69% of the between-person (i.e., Level-2) variance, and 1.12% of the within-person (i.e., Level-1) variance (ICC = 0.41).

**Table S10. GLMMs predicting perceived shared fate with all of humanity.**

|  | COVID prevalence | | | | Perceived risk | | | |
| --- | --- | --- | --- | --- | --- | --- | --- | --- |
| Fixed effects | *b* | *p* | 95% CI | | *b* | *p* | 95% CI | |
| Time | **.028** | **.017** | **.005** | **.050** | .004 | .447 | -.006 | .014 |
| *Level-2* |  |  |  |  |  |  |  |  |
| COVID prevalence | .078 | .143 | -.026 | .182 |  |  |  |  |
| Time *×* COVID | .006 | .198 | -.003 | .015 |  |  |  |  |
| Perceived risk |  |  |  |  | .067 | .112 | -.016 | .149 |
| Time *×* Risk |  |  |  |  | .010 | .086 | -.001 | .022 |
| *Level-1* |  |  |  |  |  |  |  |  |
| COVID prevalence | -.031 | .358 | -.098 | .036 |  |  |  |  |
| Time *×* COVID | -.010 | .350 | -.031 | .011 |  |  |  |  |
| Perceived risk |  |  |  |  | .011 | .694 | -.044 | .066 |
| Time *×* Risk |  |  |  |  | .002 | .590 | -.005 | .009 |
| Random effects | 𝝉 | *SE* | *p* |  | 𝝉 | *SE* | *p* |  |
| Intercept | 1.655 | .083 | <.001 |  | .769 | .038 | <.001 |  |
| Time | .005 | .0007 | <.001 |  | .010 | .001 | <.001 |  |
| COVID prevalence | .119 | .023 | <.001 |  |  |  |  |  |
| σ^2^ | 20950.13 | | |  | 20879.99 | | |  |

*Note.* COVID-19 prevalence model (Obs. = 8802, *N* = 932), Perceived risk of infection model (Obs. = 8466, *N* = 988), ICC = 0.41. Numbers in bold show statistically significant fixed effects. Perceived shared fate with humanity was measured as *All of humanity and I rise and fall together* (1 = *do not agree at all*, 7 = *strongly agree*).

**Table S11. Summary of the changes in perceived interdependence from March to August 2020.**

| Perceived interdependence | Baseline  *M*(*SD*) | Cumulative change by August 2020 | COVID-19 prevalence at baseline | Perceived infection risk at baseline | Time × COVID-19 prevalence | Time × perceived infection risk |
| --- | --- | --- | --- | --- | --- | --- |
| Emotional shared fate: When my **neighborhood** succeeds, I feel good | 4.40  (1.59) | *b* = .145 [.040, .249]** | -- | *Level-1*  *b* = .029 [.003, .055]* | *Level-1*  COVID (-1SD)  *b* = .054 [.010, .098]*  COVID (+1SD)  *b* = -.012 [-.025, -.00004] | -- |
| Perceived shared fate:  My **neighborhood** and I rise and fall together | 2.93  (1.55) | *b* = .658 [.528, .788]*** | *Level-2*  *b* = -.167 [-.272, -.063]**  *Level-1*  *b* = .376 [.304, .448]*** | -- | *Level-2*  COVID (-1SD)  *b* = .009 [-.018, .036]  COVID (+1SD)  *b* = .061 [.038, .083]***  *Level-1*  COVID (-1SD)  *b* = .072 [.030, .115]***  COVID (+1SD)  *b* = -.003 [-.015, .001] | -- |
| Emotional shared fate: When all of **humanity** succeeds, I feel good | 5.62  (1.38) | *b* = -.481 [-.590, -.371]*** | *Level-1*  *b* = -.134 [-.202, -.067]*** | -- | *Level-1*  COVID (-1SD)  *b* = .069 [.027, .111]**  COVID (+1SD)  *b* = -.013 [-.026, -.001]* | -- |
| Perceived shared fate:  All of **humanity** and I rise and fall together | 4.39  (1.75) | *b* = .125 [.017, .234]* | -- | -- | -- | -- |

*Note.* Perceived interdependence items were scored on 7-point scales (1 = *do not agree at all*, 7 = *strongly agree)*. We tested whether prevalence of COVID-19 and perceived risk of infection were associated with perceived interdependence at baseline and with changes in perceived interdependence over time. Level-2 indicates between-person effects, and Level-1 indicates within-person effects derived from GLMMs. The numbers in brackets represent 95% CIs. *** = *p* < 0.001, ** = *p* < 0.01, * = *p* < 0.05.

**S2.4. Cooperation and interdependence between neighbors and all of humanity.**

In this section we report on analyses we conducted to test whether people reported greater cooperation and interdependence with their neighbors or with all of humanity. We ran paired-sample *t*-tests between participants’ interdependence and cooperation towards neighbors and all of humanity at time 1 (i.e., beginning of this study), time 7 (i.e., midpoint of this study), and time 14 (i.e., end of this study). As shown in Table S12, people reported higher willingness to help neighbors than a person from a different country. People also reported greater need-based attitude towards helping neighbors than a person from a different country. As shown in Table S13, people reported greater emotional and perceived shared fate with all of humanity than with their neighbors.

**Table S12. Cooperation between neighbors and a person from a different country.**

| Target | Neighbors | | Humanity | |  |  |  |  |  |  |  |
| --- | --- | --- | --- | --- | --- | --- | --- | --- | --- | --- | --- |
| Inclination to cooperate | *M* | *SD* | *M* | *SD* | *t* | *df* | *p* | *M_diff_* | *95% CI* | | *d* |
| Willingness to help (T1) | 3.11 | 1.79 | 2.39 | 1.61 | 9.147 | 300 | <.001 | 0.724 | 0.568 | 0.880 | 0.527 |
| Willingness to help (T7) | 3.12 | 1.78 | 2.83 | 1.76 | 6.20 | 664 | <.001 | 0.286 | 0.195 | 0.376 | 0.240 |
| Willingness to help (T14) | 2.98 | 1.82 | 2.77 | 1.77 | 5.785 | 665 | <.001 | 0.212 | 0.140 | 0.284 | 0.224 |
| Need-based attitude (T1) | 5.64 | 1.39 | 5.42 | 1.38 | 2.833 | 302 | 0.005 | 0.228 | 0.070 | 0.386 | 0.163 |
| Need-based attitude (T7) | 5.13 | 1.45 | 4.92 | 1.63 | 5.071 | 663 | <.001 | 0.206 | 0.126 | 0.286 | 0.197 |
| Need-based attitude (T14) | 5.01 | 1.59 | 4.84 | 1.67 | 4.564 | 667 | <.001 | 0.168 | 0.096 | 0.240 | 0.177 |

**Table S13. Perceived interdependence between neighbors and all of humanity.**

| Target | Neighbors | | Humanity | |  |  |  |  |  |  |  |
| --- | --- | --- | --- | --- | --- | --- | --- | --- | --- | --- | --- |
| Perceived interdependence | *M* | *SD* | *M* | *SD* | *t* | *df* | *p* | *M_diff_* | *95% CI* | | *d* |
| Emotional shared fate (T1) | 4.40 | 1.59 | 5.63 | 1.38 | -11.755 | 301 | <.001 | -1.222 | -1.426 | -1.017 | -0.676 |
| Emotional shared fate (T7) | 4.65 | 1.61 | 5.40 | 1.45 | -14.583 | 663 | <.001 | -0.750 | -0.851 | -0.649 | -0.566 |
| Emotional shared fate (T14) | 4.67 | 1.58 | 5.28 | 1.44 | -11.640 | 666 | <.001 | -0.609 | -0.711 | -0.506 | -0.451 |
| Perceived shared fate (T1) | 2.93 | 1.55 | 4.39 | 1.76 | -12.375 | 300 | <.001 | -1.455 | -1.687 | -1.224 | -0.713 |
| Perceived shared fate (T7) | 3.62 | 1.73 | 4.53 | 1.71 | -14.004 | 662 | <.001 | -0.905 | -1.032 | -0.778 | -0.544 |
| Perceived shared fate (T14) | 3.81 | 1.73 | 4.66 | 1.64 | -13.677 | 663 | <.001 | -0.855 | -0.978 | -0.733 | -0.530 |
