# Supplementary material for: Shared fate was associated with sustained cooperation during the COVID-19 pandemic
Source: PLoS One. 2024 Sep 26;19(9):e0307829. doi: 10.1371/journal.pone.0307829 (PMC11426462; doi:10.1371/journal.pone.0307829)
Supplement: Attachment — Submitted filename: Cooperation in pandemic - response to reviewers - plos.docx [file pone.0307829.s002.docx]

Dear Dr. Horita,

My coauthors and I are grateful for the opportunity to revise our manuscript, and submit it for reconsideration at *PLOS ONE*. We would like to thank you and the reviewers for taking the time to read our manuscript and provide such thorough, thoughtful, and constructive comments. We have read the reviews carefully, and have made substantial revisions addressing the concerns expressed. Most importantly, (1) we included a measure of COVID-19 prevalence and re-ran our analyses to include this additional covariate. (2) We addressed reviewers’ concerns regarding citations and nomenclature, (3) as well as reviewer’s concerns with sample characteristics and measures employed. We’re especially grateful with the reviewers’ suggestion to include cases of COVID-19 to our analysis. We are confident our paper now presents a more complete, nuanced, and interesting account of people’s inclinations to cooperate and perceived interdependence during the early period of the pandemic.

Below we provide a consolidated point-by-point summary of reviewers’ concerns, explaining how (and where) we addressed them in the revised manuscript. To ease the readability of this document, editor and reviewer comments are shown in bold, our responses are shown below, and changes to the manuscript are shown indented and italicized. We are optimistic that our revisions have sufficiently addressed each of the reviewers’ concerns, questions, and suggestions, and we appreciate the ways in which the paper has improved as a result. We look forward to receiving your assessment of the revised manuscript in due time.

Best,

Diego Guevara Beltran, PhD

**Editor comments**

1. **Regarding the description of Figure 4(c), I think there seems to be a discrepancy.**

We thank the editor for noticing this oversight. We have redone our graphs and made sure the labels properly represent the statistical results.

2. **Citations**

We edited the in-text citations and reference section to Vancouver style.

3. **Formatting**

We edited the manuscript to comply with formatting requirements.

**REVIEWER 1**

1. **The study uses single-item measures, with no indication of their origin, reliability or validity. I assume that this decision was taken to make the questionnaire more acceptable to respondents? The items seem intuitive so they are probably mostly ok (the authors themselves acknowledge that one of the measures of social support is seriously ambiguous), but I would have expected some explanation and justification of them in the method section and an explicit acknowledgment of the limitations of them in the discussion.**

We included the following text in the method section to address the reviewer’s concern about the items:

*Depending on the setting and source, fitness interdependence can manifest psychologically through perceptions of interdependence such as perceived similarity in the context of relatedness or kin relationships, via self-other-merging in the context of intergroup or large scale disasters; and relationship closeness in the context of romantic relationships. At their core, these psychological manifestations of fitness interdependence track the extent to which other’s outcomes are likely to influence one’s own outcomes. The shared fate scale was designed to measure perceptions of interdependence globally (i.e., across settings, sources, and relationships), and is a reliable measure comprised of two subscales that have shown concurrent, discriminant, convergent, and predictive validity (32).*

*Perceived shared fate (e.g., [target] and I rise and fall together) tracks beliefs about the extent to which partners’ rewarding and aversive outcomes are likely to translate into a personal reward or loss. For example, extent to which people believe a partner’s job promotion will have a positive influence on one’s life, or extent to which people believe a partner’s parents’ death will have a negative influence on one’s life. Emotional shared fate (e.g., when [target] succeeds, I feel good) tracks affective responses to partners’ outcomes. Specifically, the intensity of emotional shared fate corresponds to the appraisals people make about the extent to which others’ positive and negative outcomes are likely to translate into a personal reward or loss. For example, how good one feels when one’s partner receives a job promotion, or how bad one feels when said partner’s parents pass away (32).*

*Perceived and emotional shared fate inform people about the extent to which they are positively interdependent with specific relationship partners, in turn predicting how much to value such partners, and how much to invest in their welfare (32,35). Although the shared fate scale was developed as a six-item measure, here we employed only one item per subscale to reduce participant fatigue, and maximize the allocation of time and resources for other survey items. We selected the item “when [target] succeeds, I feel good” as the index of emotional shared fate because at Time 1, this item showed the strongest correlation with need-based helping attitude towards neighbors (r = 0.35, p < 0.001), compared to other items (r’s = 0.07 to 0.28). Similarly, we selected the item “[target] and I rise and fall together” as the index of perceived shared fate because at Time 1, this item showed the strongest correlation with willingness to help a person from a different country (r = 0.24, p < 0.001), compared to the other items (r’s = 0.01 to 0.22).*

In the limitations section, we discuss limitations of our measures, including that the willingness to help item could have indexed a tradeoff between disease avoidance and willingness to help; as well as limitations regarding our shared fate items, including the fact that the shared fate scale was designed to measure interdependence at the level of relationships, rather than situations, and that perceived shared fate could have indexed both perceptions of negative and positive interdependence.

2. **The other issue is I was slightly surprised that there was no account of what was happening with the pandemic in the period in question. Surely participant answers on questions concerning common fate and helping will be affected by whether there is a wave.**

We thank the reviewer for making this excellent suggestion, we reanalyzed the data including incidence of COVID-19 infections. We report updated analyses in the main text and supplements. We also include this text in the method section:

*To determine the local prevalence of COVID-19, we matched participants’ location information to the COVID-19 Data Repository maintained by the Center for Systems Science and Engineering (CSSE) at Johns Hopkins University (9). Using this database, we used the latitude and longitude of each participants’ postal code and matched the postal codes to the corresponding location in the COVID-19 Data repository to determine cumulative incidence rates of COVID-19 (i.e., confirmed cases per 100,000 individuals) of 520 participants. We were not able to match the prevalence of COVID-19 for participants who did not provide postal codes or provided unidentifiable postal codes.*

3. **p. 3/11 of the pdf ‘Sometimes people become fearful and aggressive, stealing resources and committing crimes (Drury et al., 2013). This is an odd choice of reference given that the paper cited is about disaster myths.**

We apologize for this oversight, we have removed this citation and replaced it for a paper showing both decreases (at the local county level), and increases (in the surrounding areas) in criminal activity following disasters (1).

4. **p. 3/11 of the pdf ‘Crises can create positive interdependence, eliciting perceptions of shared fate and promoting cooperation (e.g., Andrighetto et al., 2016; Drury et al., 2016)’ The Drury et al. study found no direct relation between common fate and cooperation. In line with the social identity approach, common fate predicted shared identity and it was shared identity that predicted cooperation.**

We edited this sentence:

*Crises can create positive interdependence, eliciting perceptions such as shared fate and shared identity, and in turn promoting cooperation (3,4).*

5. **p. 4/ 12 of the pdf ‘and panic buying were prevalent aspects of behavior (The White House, 2020)’. I don’t know any source suggesting that so called panic buying was ‘prevalent’ during the pandemic. The excellent study by Kantar showed that just a small number of people bought extra, and that only a minority of these bought very large amounts. Moreover these episodes of extra buying occurred at the start of the pandemic rather than operating throughout 2020. The source cited (The White House) was ‘page not found’ so I couldn’t check what it says.**

We have corrected this sentence:

*At the same time, more people were purchasing household goods (e.g., cleaning supplies, non-perishable foods) during March 2020 compared to the previous year (18), fueling the belief that people were looking out for themselves (e.g., hoarding) and incentivizing others to behave similarly (19). Such fears, while factually unfounded (18), even led the secretary of the United States to take action to mitigate the potential risk of hoarding and price gouging (20).*

The executive order referenced above can be found here: https://trumpwhitehouse.archives.gov/presidential-actions/executive-order-preventing-hoarding-health-medical-resources-respond-spread-covid-19/

6. **p. 8/16 of the pdf ‘After a natural disaster (i.e., flood, earthquake)’ Some argue that the term ‘natural disasters’ is misleading: see** [**https://www.nonaturaldisasters.com/**](https://www.nonaturaldisasters.com/)

We deleted “natural” in all mentions of disasters.

7. **p. 18 of the pdf ‘For example, cooperation could marginally increase over time, as has been reported in most crises.’ This claim is not referenced. Also, in the readings I’m more aware of the opposite is the case. Immediately after a disaster there is extensive cooperation, which then declines afterwards as people run out of resources. Krys Kaniasty has written extensively about this and is probably worth including. For application of his approach to cooperation during Covid (and for literature and data on decline in social support over the first year of the pandemic, see: Ntontis, E., Fernandes-Jesus, M., Mao, G., Dines, T., Kane, J., Karakaya, J., Perach, R., Cocking, C., McTague, M., Schwarz, A., Semlyen & J. & Drury, J. (2022). Tracking the nature and trajectory of social support in Facebook mutual aid groups during the COVID-19 pandemic. International Journal of Disaster Risk Reduction.** [**https://doi.org/10.1016/j.ijdrr.2022.103043**](https://doi.org/10.1016/j.ijdrr.2022.103043)

We thank the reviewer for pointing us in the direction of Kaniasty. His work indeed is very relevant. We included the following text citing his research:

*In parallel to volunteer rates following terrorist attacks, people who experienced greater losses during a flood received help shortly after the flood, but perceptions of received support declined considerably six months after the flood (75,76). This decline in support following crises is a result of both exposure to crises, and unmet needs or expectations, rather than objective declines in received support per se. Controlling for received support, people who experienced more losses during a hurricane reported lower perceptions of received support two years after the hurricane, suggesting that unmet needs contribute to declines in perceived support regardless of objectively received support (77). Moreover, greater exposure to disasters can onset or exacerbate mental health problems (e.g., PTSD), which in the longer term (i.e., 12-18 months post disaster), can lead to steeper declines in perceptions of received support (78).*

We also include the following text citing Ntontis et al:

*Studies during the COVID-19 pandemic point to a pattern of initial increases in cooperation, followed by decreases in the longer term. For example, activity in online mutual aid groups (i.e., posts requesting and offering help) increased sharply during the first wave of the pandemic (i.e., March 2020), but declined to baseline levels by July 2020 (84).*

8. **Why this sample size?**

We were under time pressure to launch the survey (1 week) given access to research funds available at the time, as well as the need to launch the survey before plausible changes continued to accumulate. Given this time pressure, we relied on recommendations to detect two- and three-way interactions in longitudinal mixed-effects models. With 400 participants and a minimum of 8 time periods, we can confidently (i.e., with 80% power) detect time x covariate interactions of even small effect sizes. With 300-500 participants, we can also detect with confidence medium-to-large three-way interactions (He & Leon, 2010; see link below). Thus, we aimed to recruit 500 participants. <https://www.tandfonline.com/doi/full/10.1080/10543401003618819>

9. **Why the extra recruitment at time 3?**

With 20% attrition at Time 2, we decided to recruit another set of participants in hopes of obtaining 500 participants by the end of the survey.

10. **Why these particular timepoints?**

We sought to maximize the number of time points, given available funds, so that we could get a detailed account of the developmental trajectory of measures employed. Maximizing time points allowed us to (a) document the within-person variability in perceived interdependence and inclinations to cooperate over time, and (b) measure perceived infection risk and perceived interdependence as a time-varying covariate (see Figures 2-3).

11. **I can’t follow how the t1-2 data can be used with the other data.**

An advantage of mixed-effects models is the maximum likelihood estimation method. This allows the estimation of parameters even with missing data at varying time points. In addition, estimates/parameters (e.g., slope of time), are informed by all available information at level-1 (i.e., within-person observations). However, it is weighed by the information provided at the level of the cluster (i.e., the between-person level). The model can make a “best guess” of a person’s individual trajectory/slope based on all the pooled information. Just like in single-level regression, the model finds the line of best fit when looking across all individual slopes/trajectories (i.e., the fixed effects, such as the average slope of all individual slopes, or the average intercept of all individual intercepts), but then uses this information in combination with the random effects (i.e., estimates of between-person variance surrounding fixed-effects) to also make guesses/predictions about the person-specific estimates (e.g., individual-level intercepts, slopes). Below is a graphical representation of this process (see participant 373, 374 for cases with missing data; green line shows the predicted individual trajectory when “pooling” information from all individual trajectories): https://www.tjmahr.com/plotting-partial-pooling-in-mixed-effects-models/

12. **Demographics – provide age range, not just SD?**

We edited the reporting of age:

*(M_age_ = 28.65, SD_age_ = 10.23, Min_age_ = 18, Max_age_ = 80, 50.35% men).*

13. **How long did it take and how much were people paid?**

Duration per time period is reported on Table 1. We now also add the following sentence:

*Participants were compensated with $1.75 USD for Times 1-4 ($10.10 USD/hour on average), and an average of $1.50 USD ($8.64 USD/hour on average) from times 5-14.*

14. **p. 31 of the pdf ‘people high on self-other-merging, a marker of perceived interdependence’ But there wasn’t a measure of ‘self other merging’, was there?**

We apologize for the oversight, we used self-other-merging for identity fusion. We deleted the referenced sentence from the revised manuscript.

15. **p. 31 of the pdf ‘Our results have potential implications for emergency preparedness and suggest that opportunities to build interdependence within communities prior to an emergency might be important for sustaining cooperation when crises hit.’ This is the basis of many countries’ approach to community resilience, so cite some examples.**

We added the following text, citing Ntonis et al (2020):

*Indeed, a longitudinal study during the COVID-19 pandemic finds that shared identity with one’s community at baseline predicted future perceived support, but perceived support did not predict future shared identity (90).*

16. **Why is table 3 in the discussion?**

We moved Table 3 to the end of the results, immediately before the discussion.

**Reviewer 2**

1. **Under such an unknown and ongoing crisis, some people would have acted as supporters or selfishly according to "social norms" such as government guidelines and local community rules. Simple dichotomous statements such as supportive and selfish people (Lines 85 to 100) risk oversimplifying the discussion.**

*We do not mean to imply that people are either selfish or cooperative, but rather that both behaviors could be observed, as is often the case during times of crises (1). In addition, given the high levels of uncertainty posed by a novel pandemic, people’s cooperative or selfish behaviors were largely influenced by locally relevant descriptive norms (25).*

2. **Lines 140-160: Request for causal clarification of arguments based on fitness interdependence theory. This paragraph suggests that fitness interdependence theory may go beyond interrelationships among individuals and within relatively small groups to the larger group level. If my understanding is correct, why not cite and organize one of the clues, "a history of sharing" (Line 122), to make it clearer to the reader? I was under the impression that the statement in this paragraph is the reverse of the causal relationship with the fitness interdependence theory. That is, in the fitness interdependence theory, I understood that the three clues in line 114 exist as causes, which in turn affect each other in terms of disaster survival (lines 133 to 139). However, from lines 140 to 160, it seemed to me that there is another process of acquiring an interdependent perception of the experience of disasters such as earthquakes and terrorist attacks as cues.**

There are four key terms that underlie our argument here.

1) Fitness interdependence: the extent to which one’s outcomes are correlated with one’s partner’s survival, reproduction, and wellbeing –and vice-versa.

2) Settings of fitness interdependence: these are contexts or situations in which we can reliably observe that people are, or will become, interdependent. These settings include things like mating/reproduction, marriage/affine relationships, alloparenting, co-residence, mutual support, group membership, and intergroup conflict.

3) Cues/sources of perceived interdependence: these describe the person’s attributes (e.g., relatedness, phenotypic similarity), or situational experiences (e.g., history of sharing) that reliably induce perceptions of interdependence.

4) Perceived interdependence: the psychological manifestation of fitness interdependence. These psychological manifestations of fitness interdependence might be specific to a setting of fitness interdependence (e.g., perceived similarity as a cue to relatedness; self-other-merging as a cue to group membership), they may overlap (e.g., history of sharing with one’s kin, with one’s affines, with one’s group), or they may be global (e.g., perceptions of shared fate with specific partners not taking into account setting or specific cues).

The theorized causal relationship among these variables is as follows: setting(s) of fitness interdependence -> cue(s) or source(s) of perceived interdependence -> perceived interdependence. The process that involves experiences of disasters inducing perceptions of interdependence is not a different process. We argue that disasters, like other settings (e.g., intergroup conflict), can be thought of as a setting of fitness interdependence *in so far as* the context generates conditions or situations in which people’s outcomes become correlated, this fitness interdependence in turn elicits perceptions of interdependence (e.g., self-other-merging with one’s group, shared fate).

3. **Line 161: Request for additions regarding the characteristics of the COVID-19 pandemic. Unlike its other crises, the COVID-19 pandemic did not initially have robust guidelines for crisis management or clear-cut right answers for cooperative behavior. Also, unlike other crises that were localized in time and geography, this pandemic was a dynamic, ongoing global crisis. I ask that you consider adding that this was a dynamic, highly unknowable, and endlessly stressful situation in the manuscript.**

We completely agree with the reviewer on this point. We have edited this section so now it reads:

*Exposure to a collective threat and experiencing opportunities to engage in mutual support are shared features between previous crises and the COVID-19 pandemic. However, unlike previously studied crises, the pandemic brought the additional challenges associated with the transmissibility of a highly infectious virus. Moreover, unlike other crises in which the risks are localized in time and space (e.g., earthquakes, floods, terrorist attacks), the COVID-19 pandemic continues to be a global and dynamic crisis, initially characterized by high levels of uncertainty and stress-inducing events (57,58). The novelty and uncertainty surrounding the pandemic also contributed to a lack of clear guidelines for collective behavior (e.g., mask wearing) during the early periods of the pandemic (59). And even when clear guidelines for collective behavior became widespread, adherence to such guidelines varied widely (25,60–62). The transmissibility of disease and general uncertainty surrounding the pandemic posed a challenge to people’s ability to forge perceived interdependence and cooperation because the risk of infection and the risk of defection can give rise to negative fitness interdependence (e.g., becoming infected when helping a person in need, helping an exploitative person).*

4. **Please note that the comparison with the terrorist incidents (i.e., localized crises) cannot be made simply (Lines 191 to 200).**

We added the following text to recognize this important point:

*To understand whether risk of infection and shared fate influenced cooperation over time during the COVID-19 pandemic, we first need to understand how these constructs develop over time during crises. While we recognize that pandemics differ meaningfully to other crises (e.g., transmissibility of disease, localization, duration), these previous studies can provide general clues as to how people might respond over time.*

5. **Line 346: Request for clarification regarding cluster-mean score. How was the individual baseline handled when calculating the cluster-mean scores?**

Apologies but we are not sure what the reviewer means here by individual baseline. Cluster mean scores are a person’s average score across all responses for a given variable (e.g., shared fate with neighbors). Thus, the average score will reflect a person’s observed responses.

**Please consider including the number of infections per day in the participant's country at each time point in the analysis model. It is possible that regional differences in the perceived infection risk at a given point in time, such as whether the infection situation in a country is severe or not, may arise.**

We thank the reviewer for making this suggestion. We have included local prevalence of COVID-19 and reanalyzed our data. Because we redid all of our analyses, we refer the reviewer to the main text of our revised manuscript.

**For results with small effects, I request careful description to prevent readers from over-interpreting them (e.g., Lines 394-395, 405-406). Also, please consider presenting a visual figure showing the 95% confidence interval.**

We edited the text to qualify the referenced effects as “small”, for example:

*A one-unit increase in time was associated with a small increase in perceived shared fate with humanity (i.e., All of humanity and I rise and fall together) (b = 0.01, SE = 0.004, CI95% [0.001, 0.02]), leading to a small increase of b = 0.12 (SE = 0.05, CI95% [0.02, 0.23]) by August 22, 2020 (SI S2.3.4; Figure 3).*

Regarding the 95% CIs, Figures 2-3 do show the 95% CIs. We now include the following text in the figure captions to make this clearer: *Shaded bands show 95% CIs.*

6. **4-1. Line 580: Request for additional prior studies. Imada & Mifune (2021) suggest that when an infectious disease is prevalent in a group, the behavior of avoiding other members of the inner group may increase (data not verified). Please consider bolstering your argument through the following and other papers. Imada, H., & Mifune, N. (2021). Pathogen threat and in-group cooperation. Frontiers in Psychology, 12, 2568.** [**https://doi.org/10.3389/fpsyg.2021.678188**](https://doi.org/10.3389/fpsyg.2021.678188)

We thank the reviewer for pointing us to the work of Imada & Mifune. We added the following text to recognize the alternative explanation provided by Imada & Mifune:

*An alternative explanation to consider here, hypothesized by Imada and Mifune (91), and empirically demonstrated by Ko et al. during the pandemic (72), is the possibility that people exert distance (e.g., avoidance, derogation behaviors) towards ingroup targets when such targets are believed to be sources of infection (72,102–104). Thus, rather than increasing shared fate towards more distant targets (i.e., all of humanity), results might reflect decreasing shared fate with closer targets (i.e., neighbors). Our results are consistent with the idea that perceived infection risk can motivate avoidance toward ingroup targets when such targets are perceived to be sources of infection, but not decreasing shared fate with ingroup members.*

*Consistent with the first hypothesis, we found that willingness to help neighbors decreased at a faster rate over time for people with high trait perceived infection risk than for people with low trait perceived infection risk, while need-based helping attitude toward neighbors decreased over time only for people with high trait perceived infection risk. However, perceived infection risk was not associated with changes in cooperation with a person from a different country. Challenging the hypothesis that disease avoidance motivation led to decreasing shared fate with the ingroup, we found that people reported higher perceived interdependence with both neighbors and humanity when they experienced lower COVID-19 prevalence. Moreover, people only reported lower perceived interdependence with humanity, not neighbors, when they experienced higher COVID-19 prevalence.*
